# An overview of randomized phase III clinical trials of cancer nanomedicines

**DOI:** 10.1016/j.cpt.2024.10.001

**Published:** 2024-10-28

**Authors:** Micael N. Melo, Ricardo G. Amaral, Lucas R. Melo de Andrade, Patricia Severino, Cristina Blanco-Llamero, Luciana N. Andrade, Eliana B. Souto

**Affiliations:** a Department of Medicine, Federal University of Sergipe, Lagarto, Sergipe 49400-000, Brazil; b Department of Physiology, Federal University of Sergipe, São Cristóvão, Sergipe 49100-000, Brazil; c Laboratory of Pharmaceutical Technology, Federal University of Mato Grosso do Sul, Campo Grande, Mato Grosso do Sul, 79070-900, Brazil; d Institute of Technology and Research, Tiradentes University, Farolândia, Aracaju, Sergipe, 49032-490, Brazil; e Laboratory of Pharmaceutical Technology, Faculty of Pharmacy, University of Porto, 4050-313 Porto, Portugal; f Faculty of Health Sciences, Francisco de Vitoria University, Pozuelo de Alarcón, 28223, Madrid, Spain; g UCD School of Chemical and Bioprocess Engineering, University College Dublin, Belfield, Dublin 4, D04 V1W8, Ireland

**Keywords:** Antineoplastic drugs, Chemotherapy, Nanomedicine, Drug delivery systems, Personalized medicine, Clinical trials

## Abstract

**Background:**

Cancer therapy has undergone significant advances in recent decades attributed to personalized medicine and targeted drug delivery. Among the promising approaches, the use of nano-based delivery systems has become a relevant approach capable of improving treatment by releasing antineoplastic drugs at the target site, improving therapeutic efficacy, minimizing cytotoxicity in healthy tissues, and ultimately, reducing the intensity of adverse effects of chemotherapy. This study prospectively evaluated the impact of formulating anti-neoplastic drugs as nanomedicines on clinical response, overall survival, safety, and quality of life of cancer patients, based on the outcomes of randomized clinical trials.

**Methods:**

A literature review was carried out by systematically searching the PubMed/MEDical Literature Analysis and Retrieval System Online (MEDLINE), Excerpta Medica Database (EMBASE), and Latin American and Caribbean Health Sciences Literature (LILACS) databases for phase III clinical trials, comparing nanomedicines with conventional therapies for the treatment of various cancer types.

**Results:**

The nanomedicines analyzed were those that are approved and used in Brazil, considering the country's emerging market for advanced cancer treatments. From a total of 303 articles found, 26 articles were selected for systematic review. Studies showed that PEGylated l-asparaginase achieved a similar therapeutic effect to that of l-asparaginase, with fewer applications due to its longer half-life. Paclitaxel bound to albumin improved therapeutic efficacy as well as reduced infusion time and solvent-related toxicity of the conventional paclitaxel formulation. PEGylated liposomal doxorubicin showed better pharmacokinetics, reduced cardiotoxicity, and improved quality of life in cancer patients compared to that of free doxorubicin.

**Conclusions:**

This study reinforces the scientific evidence of the added value of nanomedicines to improve therapeutic efficacy and reduce toxicity in patients under chemotherapy.

## Highlights

• Several antineoplastic drugs have been formulated in nanomedicines (nanodrugs) for chemotherapy.
• Clinical response, overall survival, safety, and patients' quality of life are analyzed across several databases.
• Scientific evidence supports the added value of nanomedicines in enhancing therapeutic efficacy and reducing toxicity.

## Graphical abstract

**Figure:**
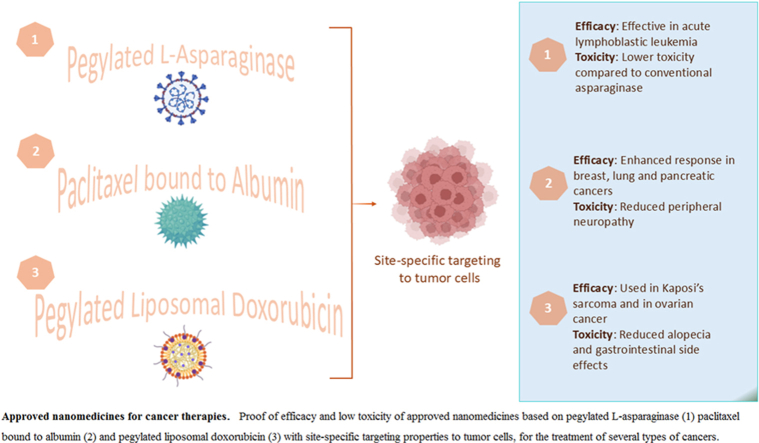

## Introduction

Cancer is a major health concern and is one of the leading causes of death worldwide.^1^ Its multifactorial origin, diverse presentation, and potential lethality make it one of the most challenging diseases for science and medicine. Despite advances in tumor biology and diagnosis, cancer mortality continues to increase annually.^2^, ^3^, ^4^

Conventional chemotherapeutic agents constitute a large proportion of cancer treatment. However, their use is limited by undesirable properties, including a narrow therapeutic window and low specificity at the site of action following oral or intravenous administration, leading to cytotoxicity in healthy tissues and contributing to the main adverse effects of this therapy.^5^

Thus, developing and improving innovative therapeutic approaches that enhance treatment effectiveness, selectively target diseased areas, and promote gradual release of the chemotherapeutic agent, thereby reducing toxicity to healthy tissues, have become instrumental in both basic and translational oncology research.^6^, ^7^, ^8^ Nanoparticle-based formulations have emerged as a promising platform for modulating drug pharmacokinetics, and have been approved worldwide for antineoplastic therapy.^9^

Nanoparticles were first successfully employed in the 1990s to improve the pharmacological treatment of malignant neoplasms. Since then, these advancements have led to the approval of numerous nanopharmaceuticals and continue to drive ongoing research, with global investment projected to reach $350.8 billion by 2025.^10^ The first nanomedicine to receive clinical approval was the PEGylated liposomal formulation of doxorubicin in 1995 (liposomal doxorubicin hydrochloride PEGylated liposomal doxorubicin [PLD]). Following this breakthrough, more than a dozen nanopharmaceuticals have been developed and brought to market,^11^ offering improvements, such as reduced dosing frequency, extended drug retention in specific tissues, minimized drug concentration fluctuations, and enhanced therapeutic efficacy.^12^ These advantages position nanomedicines as a more convenient and innovative means for anti-tumor therapy.^4^^,^^9^^,^^13^^,^^14^

### Challenges in the translation of nanomedicines

Translating nanomedicines from laboratory to clinical practice involves addressing several critical aspects and the challenges related to drug development, regulatory approval, and clinical application.^15^^,^^16^ These aspects ensure that nanomedicines are safe, effective, and capable of being reliably produced and administered to patients.^8^^,^^11^^,^^17^^,^^18^ These challenges include:

• *Preclinical evaluation*: Conduct comprehensive studies to assess safety, toxicity, and efficacy in animal models before moving to clinical trials.
• *Scalability and manufacturing*: Ensure reproducible and high-quality manufacturing processes, being compliant with the good manufacturing practices (GMPs) to maintain product consistency and safety.
• *Regulatory approval*: Nanomedicines must meet specific regulatory guidelines and undergo rigorous clinical trials to demonstrate safety and efficacy in humans.
• *Clinical translation*: Focus on targeting, biodistribution, and administration routes to optimize therapeutic benefits.
• *Ethical and societal considerations*: Ensure patient safety, affordability, and public acceptance through clear communication and ongoing monitoring.

*Post-market surveillance*: Monitor long-term safety and effectiveness, addressing any rare or delayed adverse effects.

Conversely, while nanomedicines offer significant advantages, they also entail several drawbacks and limitations that must be addressed to maximize their potential.^19^ These limitations highlight the need for ongoing research, careful regulatory oversight, and interdisciplinary collaboration to address the challenges associated with nanomedicines,^20^, ^21^, ^22^ as follows:

a. *Complexity in manufacturing and scalability*
  • *Reproducibility issues*: Consistently producing nanomedicines at a large scale poses challenges. Variations in nanoparticle size, shape, and drug loading can affect efficacy and safety.
  • *High production costs*: The manufacturing processes for nanomedicines are typically more complex and costly than those for conventional drugs, which can limit their accessibility and affordability.
b. *Regulatory challenges*
  • *Lack of standardized guidelines*: The unique properties of nanomedicines complicate the application of standard drug evaluation guidelines, resulting in regulatory uncertainty and potential delays in approval.^10^^,^^23^
  • *Extensive safety testing*: Nanomedicines may necessitate more extensive preclinical and clinical testing to evaluate their long-term safety, particularly concerning potential toxicity and environmental impact.
c. *Toxicity and immunogenicity*^5^^,^^12^^,^^24^
  • *Unintended side effects*: Nanoparticles can sometimes accumulate in non-target tissues, leading to toxicity. Additionally, certain nanomaterials may trigger immune responses or cause unforeseen side effects.
  • *Long-term safety*: The long-term effects of nanomaterials on human health and the environment are not yet fully understood, raising concerns about their prolonged use.
d. *Challenges in drug delivery and targeting*^7^^,^^25^
  • *Limited targeting efficiency*: While nanomedicines are designed to improve drug delivery to specific sites, achieving precise targeting remains difficult. Off-target effects can still occur, reducing the overall therapeutic benefit.
  • *Biological barriers*: Nanoparticles face several biological barriers (e.g., immune system, blood–brain barrier, blood-ocular barriers) that limit their ability to reach and effectively treat the target site.
e. *Complex pharmacokinetics and pharmacodynamics*^14^^,^^25^^,^^26^
  • *Variable drug release*: The release of drugs from nanoparticles can be unpredictable, leading to variability in drug levels within the body and potential dosing issues.
  • *Altered distribution*: The pharmacokinetics of nanomedicines (how the drug is absorbed, distributed, metabolized, and excreted) and pharmacodynamics (the drug's effects in the body) differ significantly from traditional formulations, complicating dose optimization.
f. *Environmental and ethical concerns*^7^^,^^27^
  • *Environmental impact*: The production and disposal of nanomedicines may pose environmental risks due to the persistence of nanomaterials in the environment.
  • *Ethical issues*: Ethical concerns exist related to the use of nanomedicines, including issues of equity, accessibility, and the potential for unforeseen consequences due to the novel nature of these therapies.

### Market and clinical impact of nanomedicines

Nanomedicines profoundly impact the pharmaceutical market by influencing both company expenses and revenues. The complexity of developing these advanced therapies leads to significantly higher research and development (R&D) costs, as companies must invest in specialized technologies, materials, and skilled personnel.^16^^,^^17^ These increased costs are compounded by longer development timelines, stemming from the intricate nature of nanomedicines, which often require extended preclinical and clinical testing. Moreover, manufacturing nanomedicines presents substantial financial challenges, as the processes involved are more sophisticated and require rigorous quality control measures to ensure consistent production. Regulatory hurdles further increase expenses, with companies needing to conduct additional safety and efficacy studies to meet stringent approval requirements, followed by ongoing post-market surveillance. Despite these higher costs, nanomedicines can generate increased revenues through premium pricing, given their enhanced therapeutic efficacy and reduced side effects. Successful nanomedicine products can also expand a company's market share, particularly in oncology, where they offer significant advantages over conventional treatments.^7^^,^^13^^,^^18^ Additionally, reformulating existing drugs into nanomedicine versions can extend product lifecycles, maintaining or even boosting revenue as original patents expire.^20^ However, the high cost of these treatments can limit market adoption, particularly in price-sensitive markets, and pose challenges for reimbursement by healthcare systems.^11^^,^^28^ Furthermore, the complex patent landscape surrounding nanomedicines can lead to legal challenges and increased competition, potentially affecting revenues. To offset these risks, companies often engage in strategic collaborations and licensing deals, which can provide additional revenue streams and share the financial burden of nanomedicine development.^9^^,^^29^^,^^30^ Despite the challenges, investments in nanomedicines, particularly in emerging markets, can offer substantial revenue potential, making them a strategic focus for many pharmaceutical companies.^4^^,^^6^^,^^10^

Cancer treatment presents a global challenge for individual and public health, necessitating an investigation into whether nanomedicines offer an effective clinical return to patients, aligned with their pharmacotherapeutic rationale, can overcome barriers faced by conventional therapies, and can guide the search for more effective treatments against this disease.^10^^,^^30^^,^^31^ Therefore, this study aimed to conduct an integrative and systematic literature review based on clinical trials to assess the impact of incorporating nationally approved nanomedicines into antineoplastic treatment, considering the clinical outcomes of efficacy, safety, and quality of life of cancer patients. This review focused on nanomedicines that have received regulatory approval for use in Brazil, thereby providing a targeted analysis relevant to the local clinical landscape. This approach guarantees that the findings and discussions are directly applicable to the Brazilian healthcare system, where regulatory, economic, and accessibility factors may differ from those in other regions. By concentrating on these specific therapies, we aim to provide valuable insights and practical guidance for clinicians and policymakers in Brazil, facilitating the integration of these advanced treatments into standard oncological practice.

## Materials and methods

### Study design and databases

This integrative systematic review was based on a comprehensive search for scientific publications in the online databases PubMed/MEDical Literature Analysis and Retrieval System Online (MEDLINE), Excerpta Medica Database (EMBASE), and Latin American and Caribbean Health Sciences Literature (LILACS) between September 2023 and July 2024.

### Guiding question

To guide the present study, we formulated the following guiding question based on the population, intervention, control, and outcomes (PICO) framework: Does nanomedicine therapy increases the therapeutic efficacy and/or reduces the toxicity in cancer patients compared to conventional chemotherapy?

### Definition of nanomedicines

Nanomedicines were identified from recent publications,^11^^,^^14^^,^^24^ and their approval status was verified through the Brazilian National Health Surveillance Agency (*Agência Nacional de Vigilância Sanitária* [ANVISA]) using the website https://consultas.anvisa.gov.br/#/medicamentos/. Only nanomedicines with valid registration at the time of data collection were included in this meta-analysis.

### Search strategy

Given the definition of nanomedicines of interest, publications in PubMed were identified using the following search strategy: *(“Clinical trial, Phase III” OR “Clinical trials, Phase III as topic” OR “Phase III clinical trial”) AND (“Neoplasms” OR “Cancer” OR “Leukemia” OR “Lymphoma”)* *AND (“Drug Therapy” OR “Antineoplastic Agents”)* *AND (“Nanotechnology” OR “Nanomedicine” OR “Nanoparticles” OR “Nanostructures” OR “Drug Delivery Systems” OR “Liposomes” OR “PEGylated”)* *AND (“Doxorubicin” OR “Liposomal Doxorubicin”) OR (“Paclitaxel” OR “Albumin-Bound Paclitaxel” OR “nab-Paclitaxel”) OR (“Asparaginase” OR “PEGylated asparaginase” OR “Pegaspargase” OR “PEG-asparaginase”) OR (“Leuprolide” OR “Leuprorelin”) OR (“Mifamurtide” OR “Muramyltripeptide phosphatidylethanolamine”)*. The search in the EMBASE and LILACS databases used the same descriptors, with Portuguese and Spanish equivalents applied for the LILACS database. The “*Clinical Trials*” filter was used for all databases to further restrict the search.

### Inclusion and exclusion criteria

Inclusion criteria for this meta-analysis were articles referring to multicenter randomized phase III clinical trials that compared the nanotechnology-based drug/scheme (comparator group) with the traditional drug/scheme (control group), involving human participants, and published between 1990 and 2024 in English, Portuguese, and Spanish. This timeframe was selected to cover the dates of approval of nanomedicines by the central regulatory agencies. Exclusion criteria comprised all publications that did not meet the referenced inclusion criteria, those that were prematurely terminated due to insufficient numbers of participants or the occurrence of intolerable toxic effects of therapy, and those for which the full text was not accessible. PubMed was considered the primary database, and articles were excluded for duplication by comparing reports from other databases with those previously collected.

### Categorization, evaluation, and interpretation of results

The initial analysis of the selected trials involved a thorough reading of the articles, followed by the completion of a form capturing the key study characteristics. These included the identification of nanomedicine, first author, year of publication, location of the primary tumor, number of participants and randomization, intervention and comparison treatments, doses, treatment administration, and duration, primary and secondary outcomes, and reported results. The information in the articles that were not tabulated was considered hypothetically to frame and substantiate the discussion according to the objectives of this study.

## Results and discussion

The survey of chemotherapeutic nanomedicines approved by ANVISA showed the registration validity of five of these drugs: PLD, paclitaxel bound to albumin, PEGylated l-asparaginase, liposomal mifamurtide, and leuprorelin associated with poly lactic-co-glycolic acid (PLGA). The indications and data of the first approval and registration by ANVISA are listed in Table 1. Other nanomedicines not identified in the search may have either lacked approval or had their registrations canceled or expired without renewal by the Brazilian agency at the time of the data collection in this study.

**Table 1:** List of nanomedicines for cancer treatment approved by the Brazilian National Health Surveillance Agency (*Agência Nacional de Vigilância Sanitária* [ANVISA]).

Nanomedicine	Therapeutic indications	First approval time, year	Registration time by ANVISA, year	Reference
Pegylated l-asparaginase	Acute lymphoblastic leukemia	1994	2016	Keating et al.^32^
Paclitaxel bound to albumin	Breast cancer	2005	2015	Gradishar et al.^33^ Harris et al.^34^ Moreno-Aspitia and Perez^35^ Von Hoff et al.^36^
	Lung cancer	2012		
	Pancreatic cancer	2013		
Pegylated liposomal doxorubicin	Kaposi's sarcoma	1995	1998	Uziely et al.^37^ Northfelt et al.^38^ Manochakian et al.^39^ Wallrabenstein et al.^40^
	Ovarian cancer	1998		
	Breast cancer	2003		
	Multiple myeloma	2007		
Leuprorelin associated with PLGA	Prostate cancer	2002	2004	Sharifi et al.^41^
Liposomal mifamurtide	Osteosarcoma	2009	2012	MacEwen et al.^42^

PLGA: Poly lactic-co-glycolic acid.

**Figure 1:**
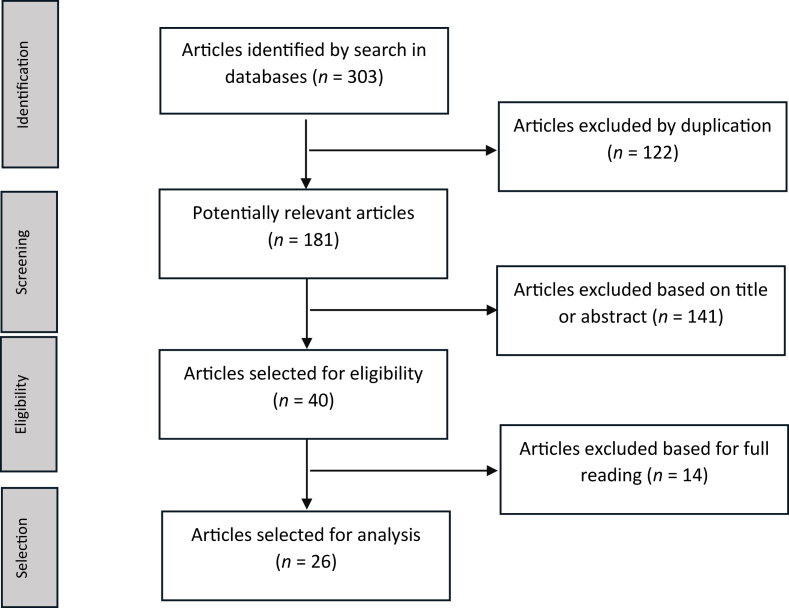
Flowchart of bibliographical research, detailing the identification, screening, and eligibility and selection of articles published between January 1990 and July 2024.

Three hundred and three potentially eligible studies were identified from the initial database searches (PubMed = 105, LILACS = 101, and EMBASE = 97), none of which were in Portuguese or Spanish. After removing duplicates, 181 articles remained. A total of 141 articles were excluded based on their titles or abstracts. The most common reasons for exclusion were that the studies were not clinical trials and did not include one of the selected nanomedicines in the comparator group. After evaluating 40 full-text articles, 14 were excluded as they were not phase III multicenter randomized controlled trials, presented preliminary results of later published full-length trials, or involved comparisons of nanomedicine use with observation, leaving 26 articles for inclusion in this study. The flowchart for article selection is shown in Figure 1.

Of the 26 articles selected, 22 focused on clinical trials of PLD, including 10 in ovarian cancer, five in multiple myeloma, four in breast cancer, and three in Kaposi's sarcoma (KS); three examined the use of paclitaxel bound to albumin, including one in breast cancer, one in lung cancer, and one in pancreatic cancer; and one addressed the benefit of PEGylated l-asparaginase in acute lymphoblastic leukemia (ALL). No clinical trials were identified that met the criteria for either leuprorelin associated with PLGA or liposomal mifamurtide; consequently, these products are excluded from the discussion of the results.

The approval of drugs typically involves several phases of clinical trials, including phase III, which are generally required to establish a medication's efficacy and safety before it can be brought to market. However, there are some nuances and exceptions to this process, especially in cases where phase III studies may not be mandatory for approval. Leuprorelin associated with PLGA and liposomal mifamurtide were approved based on clinical evidence that may have included phase II trials, prior data from related drugs, or evidence of safety and efficacy specific to their formulations.^26^^,^^43^^,^^44^ While phase III trials are generally required, regulatory agencies have provisions for using existing data, particularly for reformulations or rare diseases.^14^^,^^45^ This flexibility enables the provision of new therapeutic options while balancing the need for comprehensive evidence with practical considerations.

Leuprorelin associated with PLGA^46^^,^^47^:

• Approval and clinical trials: Leuprolide acetate is a formulation of leuprorelin associated with PLGA, a luteinizing hormone-releasing hormone (LHRH) analog used for treating prostate cancer. The original leuprolide formulation (Lupron, Abbott Laboratories, Green Oaks, IL, United States [US]) was approved based on phase III trials. Leuprolide is a depot formulation that enables extended-release of leuprolide, allowing for less frequent dosing compared to other formulations, thereby providing greater convenience for patients.
• Regulatory pathways: For extended-release formulations such as leuprorelin associated with PLGA, regulatory agencies may rely on data from the original drug's phase III trials, supplemented with additional data from studies specific to the new formulation. If the extended-release formulation demonstrates similar efficacy and safety profiles as the existing product, extensive new phase III trials might not be necessary. Instead, data demonstrating the pharmacokinetics, pharmacodynamics, and safety of the new formulation could suffice for approval.

Liposomal mifamurtide^26^^,^^48^^,^^49^:

• Approval and clinical trials: Liposomal mifamurtide is used in treating osteosarcoma, a type of bone cancer. It was approved by the European Medicines Agency (EMA) in 2009 and the U.S. Food and Drug Administration (FDA) in 2010. The approval was based on clinical trials demonstrating efficacy and safety; however, the specifics of these trials may differ from the standard phase III requirements due to the nature of the drug and the rarity of the disease it targets.
• Regulatory flexibility: For treatments of rare diseases or for drugs where traditional phase III trials pose challenges, regulatory agencies may accept alternative forms of evidence. For example, liposomal mifamurtide received approval based on a combination of clinical trial data and other forms of evidence. In some cases, drugs are approved based on phase II studies if the results are compelling and other data supports their efficacy and safety.^50^

Both the FDA and EMA have programs for accelerated approval that can allow drugs to be approved based on phase II data or other clinical evidence, particularly for serious or life-threatening conditions where few alternatives exist. This is based on the premise that the benefits outweigh the risks and that further studies will validate the drug's clinical benefit. For drugs, such as leuprorelin associated with PLGA, which are reformulations of existing drugs, the regulatory agencies might rely on prior data from the original drug and additional data specific to the new formulation. This approach can streamline the approval process while still ensuring safety and efficacy.^23^^,^^45^^,^^50^^,^^51^

**Figure 2:**
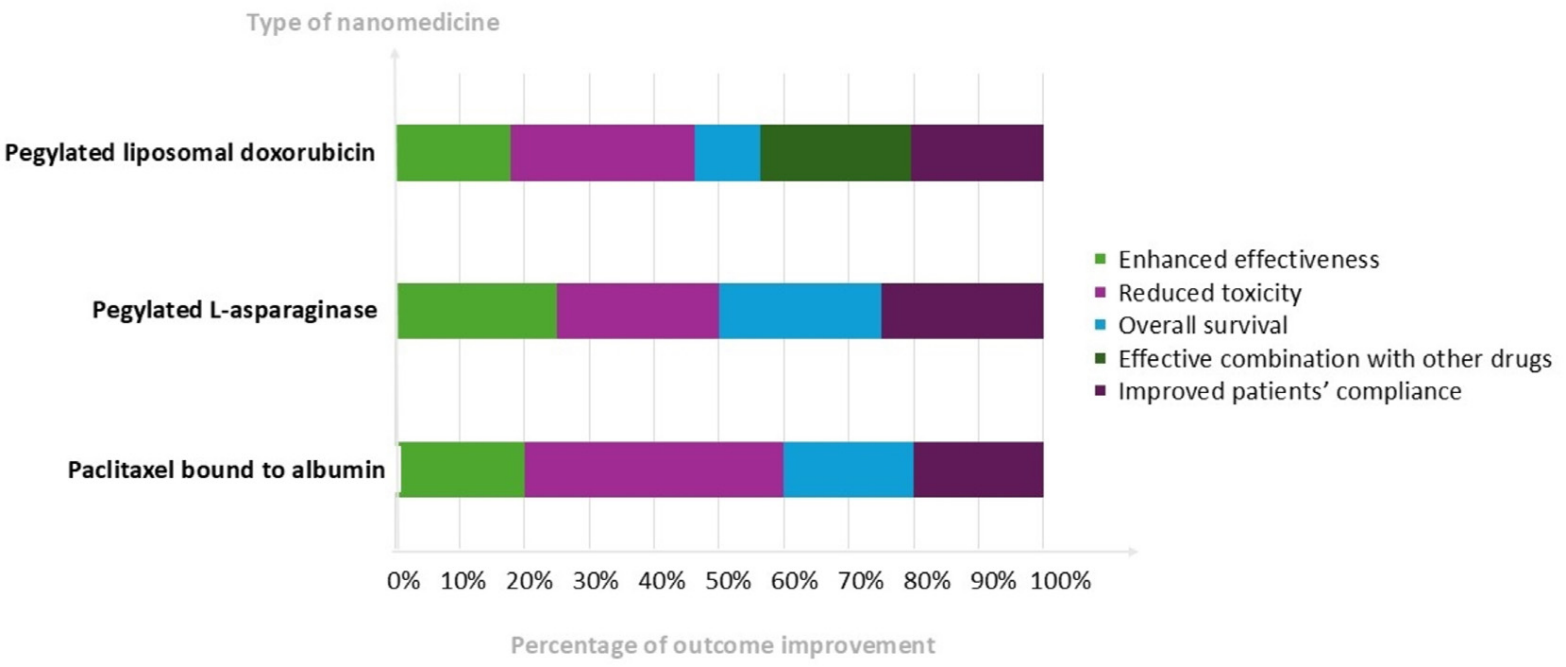
Recorded main outcomes of cancer nanomedicines described in the articles selected and discussed in Table 2, highlighting the percentage of improvements reported for the three types of nanomedicines regarding effectiveness, reduced toxicity, overall survival, efficacy in combination with other drugs, and patients' compliance.

Table 2 and Figure 2 summarize the characteristics of the clinical trials included in this systematic review, detailing the authors’ identification data, publication year, evaluated nanomedicine, cancer type, participant distribution, treatment conditions for the intervention and control groups, primary and secondary outcomes, and the main results.

**Table 2:** Summary of selected clinical trials.

Nanomedicine	Authors	Type of cancer	Randomization	Treatment	Endpoints	Study ID
PEGylated l-asparaginase	Place et al.^52^	Acute lymphoblastic leukemia	463 patients (native l-asparaginase de *E. coli*, *n* = 231; PEGylated l-asparaginase, *n* = 232)	PEGylated l-asparaginase 15 doses of 2500 IU/m^2^ IV every 2 weeks *vs. E. coli* native l-asparaginase 30 doses of 25,000 IU/m^2^ IM per week	Primary endpoint: Toxicity Secondary endpoints: Disease-free survival, nadir of serum asparaginase activity, quality of life, overall survival, and event-free survival	NCT00400946
Paclitaxel bound to albumin	Gradishar et al.^33^	Metastatic breast cancer	454 patients (paclitaxel bound to albumin, *n* = 229; paclitaxel standard, *n* = 225)	Paclitaxel bound to albumin 260 mg/m^2^ IV over 30 min without corticosteroid or antihistamine premedication *vs.* standard paclitaxel 175 mg/m^2^ IV over 3 h with premedication	Primary endpoint: Overall response rate Secondary endpoints: Time to tumor progression, overall survival, and quality of life	NCT00046527
Paclitaxel bound to albumin	Socinski et al.^53^	Advanced non-small cell lung cancer	1.052 patients (paclitaxel bound to albumin + carboplatin, *n* = 521; paclitaxel + carboplatin, *n* = 531)	Paclitaxel bound to albumin 100 mg/m^2^, 30 min infusion on days 1, 8, and 15 + carboplatin AUC 6 mg·mL^–1^·min^–1^ on day 1 every 3 weeks *vs.* paclitaxel 200 mg/m^2^ 3 h infusion + carboplatin AUC 6 mg·mL^–1^·min^–1^, both given every 3 weeks	Primary endpoint: Overall response rate Secondary endpoints: Progression-free survival, overall survival, and incidence of treatment-related adverse events	NCT00540514
Paclitaxel bound to albumin	Von Hoff et al.^8^	Metastatic pancreatic adenocarcinoma	861 patients (paclitaxel bound to albumin + gemcitabine, *n* = 431; gemcitabine, *n* = 430)	Paclitaxel bound to albumin 125 mg/m^2^ body surface area followed by gemcitabine 1000 mg/m^2^ on days 1, 8, and 15 every 4 weeks *vs.* monotherapy with gemcitabine 1000 mg/m^2^ weekly for 7 of 8 weeks (cycle 1) and then on days 1, 8 and 15 every 4 weeks (cycle 2 and subsequent cycles)	Primary endpoint: Overall survival Secondary endpoints: Progression-free survival and overall response rate	NCT00844649
PEGylated liposomal doxorubicin	Stewart et al.^54^	AIDS-related Kaposi's sarcoma	241 patients (PEGylated liposomal doxorubicin, *n* = 121; bleomycin + vincristine, *n* = 120)	PEGylated liposomal doxorubicin 20 mg/m^2^*vs.* bleomycin 15 IU/m^2^ + vincristine 2 mg every 3 weeks for six cycles	Primary endpoint: Responses to therapy (complete clinical response, partial response, progressive disease, and stable disease) Secondary endpoint: Toxicity	NCT00002105
PEGylated liposomal doxorubicin	Northfelt et al.^38^	AIDS-related Kaposi's sarcoma	258 patients (PEGylated liposomal doxorubicin, *n* = 133; doxorubicin + bleomycin + vincristine, *n* = 125)	PEGylated liposomal doxorubicin 20 mg/m^2^*vs.* doxorubicin 20 mg/m^2^ + bleomycin 10 mg/m^2^ + vincristine 1 mg every 14 days for six cycles	Primary endpoint: Responses to therapy (complete clinical response, partial response, progressive disease, and stable disease) Secondary endpoint: Toxicity	NCT00002318
PEGylated liposomal doxorubicin	Osoba et al.^55^	AIDS-related Kaposi's sarcoma	258 patients (PEGylated liposomal doxorubicin, *n* = 133; doxorubicin + bleomycin + vincristine, *n* = 125)	PEGylated liposomal doxorubicin 20 mg/m^2^*vs.* doxorubicin 20 mg/m^2^ + bleomycin 10 mg/m^2^ + vincristine 1 mg every 14 days for six cycles	Primary endpoint: Health-related quality of life	NCT00002318
PEGylated liposomal doxorubicin	Dimopoulos et al.^56^	Symptomatic multiple myeloma	259 patients (vincristine + doxorubicin + dexamethasone, *n* = 127; vincristine + PEGylated liposomal doxorubicin + dexamethasone, *n* = 132)	Vincristine 0.4 mg IV + doxorubicin 9 mg/m^2^ IV + dexamethasone 40 mg orally daily for 4 consecutive days *vs.* vincristine 2 mg IV + PEGylated liposomal doxorubicin 40 mg/m^2^ IV on day 1 + dexamethasone 40 mg orally daily for 4 days. The two regimens were administered every 28 days for four cycles, and in cycles 1 and 3, in both arms, dexamethasone was also administered on days 9–12 and 17–20	Primary endpoint: Overall response rate and toxicity	NCT02471820
PEGylated liposomal doxorubicin	Rifkin et al.^57^	Newly diagnosed multiple myeloma	192 patients (vincristine + doxorubicin + dexamethasone, *n* = 95; vincristine + PEGylated liposomal doxorubicin + dexamethasone, *n* = 97)	PEGylated liposomal doxorubicin 40 mg/m^2^ + vincristine 1.4 mg/m^2^; maximum, 2.0 mg IV on day 1 + dexamethasone 40 mg orally daily for 4 days *vs.* vincristine 0.4 mg per day + doxorubicin conventional 9 mg/m^2^ per day IV continuous on days 1–4 + dexamethasone 40 mg PO. Treatment was repeated every 4 weeks for at least four cycles	Primary endpoints: Objective response rate, toxicity, and clinical benefit Secondary endpoints: Time to disease progression, overall survival, safety, and tolerability	NCT04782687
PEGylated liposomal doxorubicin	Orlowski et al.^58^	Relapsing or refractory multiple myeloma	646 patients (bortezomib, *n* = 322; bortezomib + PEGylated liposomal doxorubicin, *n* = 324)	Bortezomib 1.3 mg/m^2^ IV on days 1, 4, 8, and 11 of a cycle every 21 days *vs.* bortezomib (1.3 mg/m^2^ IV) on days 1, 4, 8, and 11 + PEGylated liposomal doxorubicin 30 mg/m^2^ on day 4. Treatment was continued until disease progression, unacceptable treatment-related toxicity, or up to eight cycles	Primary endpoint: Time to tumor progression Secondary endpoints: Overall survival, progression-free survival, overall response rate, and safety	NCT00237627
PEGylated liposomal doxorubicin	Sonneveld et al.^59^	Relapsing or refractory multiple myeloma	646 patients (bortezomib, *n* = 322; bortezomib + PEGylated liposomal doxorubicin, *n* = 324)	Bortezomib 1.3 mg/m^2^ IV on days 1, 4, 8, and 11 of a cycle every 21 days *vs.* bortezomib (1.3 mg/m^2^ IV) on days 1, 4, 8, and 11 + PEGylated liposomal doxorubicin 30 mg/m^2^ on day 4. Treatment was continued until disease progression, unacceptable treatment-related toxicity, or up to eight cycles	Primary endpoint: Time to tumor progression Secondary endpoints: Overall survival, overall response rate, and safety	NCT00002878
PEGylated liposomal doxorubicin	Orlowski et al.^60^	Relapsing or refractory multiple myeloma	646 patients (bortezomib, *n* = 322; bortezomib + PEGylated liposomal doxorubicin, *n* = 324)	Bortezomib 1.3 mg/m^2^ IV on days 1, 4, 8, and 11 of a cycle every 21 days *vs.* bortezomib (1.3 mg/m^2^ IV) on days 1, 4, 8, and 11 + PEGylated liposomal doxorubicin 30 mg/m^2^ on day 4. Treatment was continued until disease progression, unacceptable treatment-related toxicity, or up to eight cycles	Primary endpoint: Time to tumor progression Secondary endpoints: Overall survival, progression-free survival, and overall response rate	NCT00103506
PEGylated liposomal doxorubicin	Keller et al.^61^	Taxane-refractory advanced breast cancer	301 patients (PEGylated liposomal doxorubicin, *n* = 150; vinorelbine or mitomycin C + vinblastine, *n* = 151)	PEGylated liposomal doxorubicin 50 mg/m^2^ every 28 days *vs.* vinorelbine 30 mg/m^2^ weekly or mitomycin C 10 mg/m^2^ day 1 and every 28 days + vinblastine 5 mg/m^2^ day 1, day 14, day 28, and day 42, every 6–8 weeks	Primary endpoint: Progression-free survival Secondary endpoints: Overall survival, overall response rate, duration of response, event-free survival, tolerability, health-related quality of life, and clinical benefit	NA
PEGylated liposomal doxorubicin	O'Brien et al.^62^	Metastatic breast cancer	509 women (PEGylated liposomal doxorubicin, *n* = 254; doxorubicin, *n* = 255)	PEGylated liposomal doxorubicin 50 mg/m^2^ every 4 weeks *vs.* doxorubicin 60 mg/m^2^ every 3 weeks	Primary endpoints: Progression-free survival and cardiotoxicity Secondary endpoints: Overall survival, overall response rate, tolerability, and utilization of health resources	NA
PEGylated liposomal doxorubicin	Sparano et al.^63^	Advanced breast cancer previously treated with anthracycline	751 patients (docetaxel, *n* = 373; PEGylated liposomal doxorubicin + docetaxel, *n* = 378)	Docetaxel 75 mg/m^2^*vs.* PEGylated liposomal doxorubicin 30 mg/m^2^ + docetaxel 60 mg/m^2^ every 21 days and continued until disease progression or prohibitive toxicity	Primary endpoint: Time to progression Secondary endpoints: Overall survival, objective response rate, progression-free survival, time to first response, and duration of response	NCT00091442
PEGylated liposomal doxorubicin	Harbeck et al.^64^	Metastatic breast cancer	210 patients (PEGylated liposomal doxorubicin, *n* = 105; capecitabine, *n* = 105)	PEGylated liposomal doxorubicin 50 mg/m^2^ every 28 days *vs.* capecitabine 1250 mg/m^2^ twice daily for 14 days every 21 days. Treatment continued until disease progression or unacceptable toxicity	Primary endpoint: Time to progression Secondary endpoints: Overall response rate, overall survival, time to treatment failure, quality of life, and safety	NCT01817452
PEGylated liposomal doxorubicin	Gordon et al.^65^	Recurrent and refractory epithelial ovarian cancer	474 patients (PEGylated liposomal doxorubicin, *n* = 239; topotecan, *n* = 235)	PEGylated liposomal doxorubicin 50 mg/m^2^, 1 h infusion every 4 weeks *vs.* topotecan 1.5 mg·m^–2^·d^–1^ for 5 consecutive days every 3 weeks	Primary endpoint: Time to progression Secondary endpoints: Overall survival, response rate, time to response, duration of response, safety, and toxicity	NCT00057720
PEGylated liposomal doxorubicin	Pujade-Lauraine et al.^66^	Late-relapse platinum-sensitive ovarian cancer	976 patients (PEGylated liposomal doxorubicin + carboplatin, *n* = 467; carboplatin + paclitaxel, *n* = 509)	Carboplatin AUC 5 + PEGylated liposomal doxorubicin 30 mg/m^2^ every 4 weeks *vs.* carboplatin AUC 5 + paclitaxel 175 mg/m^2^ every 3 weeks for at least six cycles	Primary endpoint: Progression-free survival Secondary endpoints: Toxicity, quality of life, and overall survival	NCT00189553
PEGylated liposomal doxorubicin	Kurtz et al.^67^	Platinum-sensitive recurrent ovarian cancer	157 patients (PEGylated liposomal doxorubicin + carboplatin, *n* = 71; carboplatin + paclitaxel, *n* = 86)	Carboplatin AUC 5 + PEGylated liposomal doxorubicin 30 mg/m^2^ every 4 weeks *vs.* carboplatin AUC 5 + paclitaxel 175 mg/m^2^ every 3 weeks for at least six cycles	Primary endpoints: Toxicity and efficacy Secondary endpoints: Severe toxicity, quality of life, and safety	NCT00189553
PEGylated liposomal doxorubicin	Pignata et al.^68^	Advanced ovarian cancer	820 patients (PEGylated liposomal doxorubicin + carboplatin, *n* = 410; carboplatin + paclitaxel, *n* = 410)	Carboplatin AUC 5 + PEGylated liposomal doxorubicin 30 mg/m^2^ every 4 weeks *vs.* carboplatin AUC 5 + paclitaxel 175 mg/m^2^ every 3 weeks for at least six cycles	Primary endpoints: Progression-free survival Secondary endpoints: Overall survival, treatment activity, toxicity, and quality of life	NCT00326456
PEGylated liposomal doxorubicin	Joly et al.^69^	Recurrent ovarian cancer	976 patients (PEGylated liposomal doxorubicin + carboplatin, *n* = 467; carboplatin + paclitaxel, *n* = 509)	Carboplatin AUC 5 + PEGylated liposomal doxorubicin 30 mg/m^2^ every 4 weeks *vs.* carboplatin AUC 5 + paclitaxel 175 mg/m^2^ every 3 weeks for at least six cycles	Primary endpoint: Progression-free survival Secondary endpoint: Hypersensitivity reactions	NCT00189553
PEGylated liposomal doxorubicin	Brundage et al.^70^	Ovarian cancer	976 patients (PEGylated liposomal doxorubicin + carboplatin, *n* = 467; carboplatin + paclitaxel, *n* = 509)	Carboplatin AUC 5 + PEGylated liposomal doxorubicin 30 mg/m^2^ every 4 weeks *vs.* carboplatin AUC 5 + paclitaxel 175 mg/m^2^ every 3 weeks for at least six cycles	Primary endpoint: Health-related quality of life	NCT00538603
PEGylated liposomal doxorubicin	Gladieff et al.^71^	Partially platinum-sensitive ovarian cancer	344 patients (PEGylated liposomal doxorubicin + carboplatin, *n* = 183; carboplatin + paclitaxel, *n* = 161)	Carboplatin AUC 5 + PEGylated liposomal doxorubicin 30 mg/m^2^ every 4 weeks *vs.* carboplatin AUC 5 + paclitaxel 175 mg/m^2^ every 3 weeks for at least six cycles	Primary endpoint: Progression-free survival Secondary endpoint: Security	NCT01281254
PEGylated liposomal doxorubicin	Wagner et al.^72^	Platinum-sensitive ovarian cancer	976 patients (PEGylated liposomal doxorubicin + carboplatin, *n* = 467; carboplatin + paclitaxel, *n* = 509)	Carboplatin AUC 5 + PEGylated liposomal doxorubicin 30 mg/m^2^ every 4 weeks *vs.* carboplatin AUC 5 + paclitaxel 175 mg/m^2^ every 3 weeks for at least six cycles	Primary endpoint: Progression-free survival Secondary endpoints: Overall survival and safety	NCT00538603
PEGylated liposomal doxorubicin	Mahner et al.^73^	Platinum very sensitive ovarian cancer	259 patients (PEGylated liposomal doxorubicin + carboplatin, *n* = 128; carboplatin + paclitaxel, *n* = 131)	Carboplatin AUC 5 + PEGylated liposomal doxorubicin 30 mg/m^2^ every 4 weeks *vs.* carboplatin AUC 5 + paclitaxel 175 mg/m^2^ every 3 weeks for at least six cycles	Primary endpoint: Progression-free survival Secondary endpoints: Overall survival and safety	NCT00189553
PEGylated liposomal doxorubicin	Pfisterer et al.^74^	Platinum-sensitive recurrent ovarian cancer	682 patients (carboplatin + PEGylated liposomal doxorubicin + bevacizumab, *n* = 345; carboplatin + gemcitabine + bevacizumab, *n* = 337)	Bevacizumab 15 mg/kg, day 1 + carboplatin AUC 4, day 1 + gemcitabine 1000 mg/m^2^, days 1 and 8 every 3 weeks *vs.* bevacizumab 10 mg/kg, days 1 and 15 + carboplatin AUC 5, day 1 + PEGylated liposomal doxorubicin 30 mg/m^2^, day 1 every 4 weeks for six cycles, both followed by maintenance bevacizumab 15 mg/kg every 3 weeks in both groups	Primary endpoint: Progression-free survival Secondary endpoints: Overall survival, progression-free survival, quality of life, safety, and tolerability	NCT01837251

AIDS: Acquired immune deficiency syndrome; AUC: Area under the curve; *E. coli*: *Escherichia coli*; IM: Intramuscular; IU: International unit; IV: Intravenous; NA: Not applicable; no: Number; PO: *Per os*.

### Approved nanomedicines

#### PEGylated l-asparaginase

l-asparaginase is a naturally occurring enzyme expressed by microorganisms, and was the first approved therapeutic enzyme with antineoplastic properties, serving as a universal tool for the treatment of ALL in pediatric patients.^75^ PEGylated *Escherichia coli* l-asparaginase (PEGylated l-asparaginase or polyethylene glycol [PEG]-asparaginase) was developed to overcome some of the limitations of native *E. coli* l-asparaginase, such as short half-life, immunogenicity, and the need for intramuscular administration; structurally, it is a tetrameric enzyme covalently conjugated to monomethoxy polyethylene glycol (mPEG).^28^ The FDA approved it in 1994 as an alternative for patients with hypersensitive reactions to l-asparaginase and later in 2006 as a first-line treatment for ALL.^28^

##### PEGylated l-asparaginase for the treatment of acute lymphoblastic leukemia

The Dana-Farber Cancer Institute Acute Lymphoblastic Leukemia Consortium Protocol 05-001 (DFCI 05-001) compared the relative toxicity and efficacy of PEGylated l-asparaginase and native l-asparaginase in 463 children and adolescents newly diagnosed with ALL between 2005 and 2010 in 11 centers in the US and Canada.^52^ The results indicated that PEGylated l-asparaginase was not associated with increased toxicity compared to the native l-asparaginase, showing overall frequencies of 28% and 26%, respectively (*P* = 0.060) and no significant difference in individual allergy frequency (*P* = 0.360). Additionally, intravenous PEGylated l-asparaginase did not demonstrate superior 5-year disease-free survival rates, with 90% (95% confidence interval [CI] = 86–94) for patients treated with intravenous PEGylated l-asparaginase compared to 89% (95% CI = 85–93) for those treated with native intramuscular l-asparaginase (*P* = 0.580).

Conversely, intravenous PEGylated l-asparaginase was associated with reduced treatment-related anxiety compared to native intramuscular l-asparaginase and offered a more convenient administration schedule. In addition, it achieved significantly higher median nadir levels of serum enzyme activity at all measured time points than native intramuscular l-asparaginase did, without increasing incidence of asparaginase-related complications, such as pancreatitis (*P* = 0.550) and bleeding or thrombotic events (*P* = 0.260). This suggests that a lower dose of PEGylated l-asparaginase may offer similar efficacy at a reduced cost. Thus, the authors recommended intravenous PEGylated l-asparaginase as the first-line treatment for the pediatric population with ALL.^52^

PEGylated l-asparaginase represents a significant advancement in the treatment of ALL, particularly in pediatric populations. PEGylating l-asparaginase exhibits a prolonged half-life and reduces immunogenicity in comparison to native l-asparaginase, resulting in fewer allergic reactions and more convenient dosing schedules. This has led to its adoption as a key component of ALL treatment protocols, contributing to improved event-free survival rates.^7^^,^^52^^,^^75^ However, despite its advantages, PEGylated l-asparaginase presents challenges, particularly concerning its toxicity profile, including hepatotoxicity, pancreatitis, and thromboembolic events. Future research should focus on understanding the mechanisms underlying PEGylated l-asparaginase-related toxicities and on developing strategies to predict and mitigate these adverse effects. This could include refining dosing strategies or identifying patient subgroups at higher risk. While PEGylated l-asparaginase has simplified treatment with less frequent dosing, further studies could explore optimizing dosing schedules to balance efficacy and safety, especially in high-risk or older patient populations. Investigating the potential of PEGylated l-asparaginase in other hematological malignancies or as part of combination therapies in resistant forms of ALL, could expand its clinical utility. Identifying biomarkers for hypersensitivity or resistance to pegylated l-asparaginase could facilitate personalized treatment, ensuring that patients receive the most effective and least toxic therapies tailored to their individual risk profiles.^15^^,^^17^^,^^31^ Ongoing surveillance of long-term outcomes, particularly in pediatric patients, will be instrumental for understanding the impact of pegylated l-asparaginase on overall survival (OS) and quality of life, including its effects on late-onset toxicities and the risk of secondary malignancies.

#### Paclitaxel bound to albumin

Paclitaxel is a semi-synthetic chemotherapeutic agent from the taxane class. Its precursor is isolated from *Taxus brevifolia*, i.e., the bark of the Pacific yew tree, which is used to treat several types of cancer. Its mechanism of action is based on the stabilization of microtubules, preventing cell proliferation in metaphase/anaphase. Although it is a highly efficient chemotherapeutic drug, paclitaxel has several side effects, including peripheral neuropathy, neutropenia, myalgia, arthralgia, and hypersensitivity.^30^ Thus, the formulation of paclitaxel bound to albumin nanoparticles (*nab*-paclitaxel or paclitaxel bound to albumin) was developed to improve the therapeutic efficacy and toxicity of the drug compared to conventional paclitaxel formulations,^31^ being approved for the treatment of metastatic breast cancer (MBC) in 2005, non-small cell lung cancer (NSCLC) in 2012, and pancreatic cancer in 2013.^76^

##### Paclitaxel bound to albumin for breast cancer treatment

A multi-center (70 centers), international (five countries) phase III randomized clinical trial was conducted between 2001 and 2002 in 454 patients with MBC, comparing paclitaxel bound to albumin with standard oil-based polyethoxylated castor oil paclitaxel in terms of overall response rate (ORR), time to progression (TTP), OS, and quality of life.^33^

The study revealed that paclitaxel bound to albumin was superior to the standard of care, paclitaxel, in terms of both TTP and ORR in patients with MBC. The ORR of paclitaxel bound to albumin was superior across all patients (33% *vs.* 19%; *P* = 0.001), in patients receiving the study drug as first-line (42% *vs.* 27%; *P* = 0.029) or second-line (27% *vs.* 13%; *P* = 0.006) therapy, in patients with prior anthracycline exposure (34% *vs.* 18%; *P* = 0.002), patients aged ≤65 years (34% *vs.* 19%; *P* = 0.001), older patients (34% *vs.* 19%; *P* = ns), and patients with poor prognostic factors, including visceral tumor involvement (34% *vs.* 19%; *P* = 0.002).

The median TTP for all patients was substantially greater in the paclitaxel bound to the albumin-treated group than in the standard paclitaxel arm (23.0 *vs.* 16.9 weeks, respectively; with a hazard ratio [HR] of 0.75; *P* = 0.006). Furthermore, patients receiving paclitaxel bound to albumin as second-line or subsequent therapy, whose clinical trajectory would be minimally influenced by further therapies, experienced a notable extension in survival, with a 28% reduction in mortality risk. In the evaluation of OS, while no survival difference was observed in first-line patients, a statistically significant difference was observed in individuals receiving paclitaxel bound to albumin compared to conventional paclitaxel as second-line or subsequent therapy (56.4 *vs.* 46.7 weeks; HR = 0.73; *P* = 0.024).^33^

In terms of adverse events, patients treated with paclitaxel bound to albumin experienced significantly fewer grade 4 neutropenia (9% *vs.* 22%; *P* = 0.001), even at a significantly higher dose. However, the occurrence of grade 3 sensory neuropathy was greater with paclitaxel bound to albumin than with standard paclitaxel (10% *vs.* 2%; *P* = 0.001). Furthermore, hypersensitive reactions resulting from paclitaxel linked to albumin were not observed, despite the lack of premedication with corticosteroids or antihistamines and the reduced injection duration (30 min *vs.* 3 h). Consistent with the safety data, no difference in quality of life was observed between the two treatment cohorts, despite the elevated dosage administered to the group receiving paclitaxel bound to albumin. Thus, the authors noted that paclitaxel bound to albumin demonstrates improved efficacy and favorable safety compared to standard paclitaxel, allowing for higher doses and significantly enhancing treatment outcomes without compromising quality of life or patient safety, making it a major advancement in the treatment of MBC.^33^

##### Paclitaxel bound to albumin for lung cancer treatment

A large study conducted in six countries involving 1052 patients with NSCLC compared the efficacy and safety of paclitaxel bound to albumin *vs.* standard paclitaxel, both associated with carboplatin, between 2007 and 2009.^53^

Independent analysis of the outcomes showed that paclitaxel bound to albumin exhibited a significantly higher ORR than the standard paclitaxel (33% *vs.* 25%) with a response rate ratio of 1.313, (95% CI = 1.082–1.593; *P* = 0.005), and demonstrated efficacy in patients with squamous histology (41% *vs.* 24%) with a response rate ratio of 1.680 (95% CI = 1.27–2.221; *P* < 0.001). Moreover, paclitaxel bound to albumin demonstrated approximately 10% improvement in progression-free survival (PFS) (median 6.3 *vs.* 5.8 months; HR = 0.902; 95% CI = 0.767–1.060; *P* = 0.214) and OS (median 12.1 *vs.* 11.2 months; HR = 0.922; 95% CI = 0.797–1.066; *P* = 0.271), with statistically significant results in patients aged ≥70 years with squamous cell carcinoma in the US.^53^

In terms of treatment toxicity, paclitaxel bound to albumin showed significantly lower rates of grade 3 neuropathy, neutropenia, arthralgia, and myalgia. However, it was associated with higher instances of thrombocytopenia and anemia, which were effectively managed during the study. The authors concluded that paclitaxel bound to albumin + carboplatin regimen presents a favorable benefit–risk profile compared to standard paclitaxel as first-line treatment for all patients with advanced NSCLC.^53^

##### Paclitaxel bound to albumin for pancreatic cancer treatment

An international (11 countries), multi-center (151 centers), open-label, randomized phase III study evaluated the efficacy and safety of the combination of paclitaxel bound to albumin and gemcitabine compared to gemcitabine monotherapy in 861 patients with metastatic pancreatic cancer between 2009 and 2012.^8^ The results revealed a median OS of 8.5 months for the paclitaxel bound to albumin + gemcitabine group compared to 6.7 months in the gemcitabine group (HR for death = 0.72; 95% CI = 0.62–0.83; *P* < 0.001). The 1- and 2-year survival rates were significantly higher in the paclitaxel bound to albumin + gemcitabine group than in the gemcitabine group (35% *vs.* 22% at 1 year and 9% *vs.* 4% at 2 years, respectively). The median PFS was 5.5 months in the abraxane + gemcitabine arm compared to 3.7 months in the gemcitabine arm (HR for disease progression or death = 0.69; 95% CI = 0.58–0.82; *P* < 0.001). The ORR was significantly higher with paclitaxel bound to albumin + gemcitabine than with gemcitabine (23% [95% CI = 19–27] *vs.* 7% [95% CI = 5–10]; *P* < 0.001), as was the rate of disease control (*P* < 0.001).

The most common non-hematological adverse events of grade ≥3 reported with paclitaxel bound to albumin + gemcitabine *vs.* gemcitabine were peripheral neuropathy (17% *vs.* 1%), fatigue (17% *vs.* 7%), and diarrhea (6% *vs.* 1%). The most common treatment-emergent hematological adverse events were leukopenia (31% *vs.* 16%) and neutropenia (38% *vs.* 27%). The incidence of anemia and thrombocytopenia was similar in both groups. However, despite the higher incidence of adverse events, these appeared to be reversible over the course of the study. The results of this study therefore support the use of paclitaxel bound to albumin in combination with gemcitabine in the first-line treatment of patients with metastatic pancreatic adenocarcinoma.^8^

Paclitaxel bound to albumin has revolutionized the delivery of paclitaxel by eliminating the need for toxic solvents,^30^^,^^33^^,^^71^^,^^74^ leading to a better safety profile and enhanced delivery to tumor tissues. Its approval across multiple cancer types, including breast cancer, pancreatic cancer, and NSCLC, underscores its broad utility. The albumin-bound nanoparticle formulation improves drug solubility and enhances tumor penetration via receptor-mediated transport. While paclitaxel bound to albumin has demonstrated significant improvements in the PFS and OS in several cancers, the benefits can vary depending on tumor biology and the presence of specific resistance mechanisms. However, understanding and overcoming resistance mechanisms to *nab*-paclitaxel should be a priority, as these can limit the drug's efficacy in certain patient populations. Leveraging paclitaxel bound to albumin in combination with other chemotherapeutics, targeted therapies, or immunotherapies could enhance its efficacy and broaden its applicability.^8^^,^^18^^,^^30^ Developing predictive biomarkers for patient stratification could optimize therapy with paclitaxel bound to albumin, ensuring its use in patients most likely to benefit. Additionally, exploring alternative nanoparticle carriers or dual-drug loading strategies may further enhance drug delivery and therapeutic outcomes for paclitaxel.

#### Pegylated liposomal doxorubicin

Doxorubicin (or adriamycin) is a broad-spectrum antitumor agent of the anthracycline class that is widely used to treat several types of cancer, including breast, bladder, lung, stomach, ovarian, thyroid, multiple myeloma soft tissue sarcoma, and Hodgkin's lymphoma.^77^ However, the therapeutic benefits of doxorubicin are limited by the high number of adverse effects, particularly the high risk of cardiotoxicity.^78^

The formulation of PLD (Doxil®, Tibotec Therapeutics, a division of Ortho Biotech Products, L.P., Bridgewater, NJ, USA US) was developed by encapsulating the drug in a lipid nanoparticle (liposome) coated with mPEG to overcome the cardiotoxicity of the drug and improve its pharmacokinetic profile.^28^ Doxil® was recognized as the first nanoparticle-based anticancer drug to receive clinical approval from the EMA and FDA in 1995, for the treatment of KS associated with acquired immune deficiency syndrome (AIDS), and latter for recurrent ovarian cancer in 1998, MBC in 2003, and multiple myeloma in 2007.^24^

##### Pegylated liposomal doxorubicin for breast cancer treatment

The first randomized controlled phase III trial evaluating the effects of PLD in 301 patients with taxane-refractory advanced breast cancer compared to the rescue regimens of vinorelbine or mitomycin C + vinblastine, commonly used in this scenario, showed interesting results.^61^ The PFS, OS, and health-related quality of life (HQL) comprised the outcomes assessed by the authors. The PFS and OS were similar between the PLD and comparator (PFS: HR = 1.26; 95% CI = 0.98–1.62; *P* = 0.110; median, 2.9 months [PLD] and 2.5 months [comparator]; OS: HR, 1.05; 95% CI = 0.82–1.33; *P* = 0.710; median, 11.0 months [PLD] and 9.0 months [comparator]).

The most common adverse events in patients treated with PLD or the comparator group were fatigue (9%–20%), nausea (23%–31%) and vomiting (17%–20%), with no statistically significant differences between the groups. In addition, patients treated with PLD frequently experienced palmoplantar erythrodysesthesia (37%) and stomatitis (22%). Conversely, constipation (16%), neutropenia (14%), and neuropathy (11%) were more common with vinorelbine, while alopecia was low in both groups (3% and 5%). The authors concluded that PLD is a useful palliative treatment for women with taxane-refractory, significantly pretreated, advanced breast cancer as it has comparable efficacy to standard rescue regimens without causing significant myelosuppression and neuropathy, in addition to the convenience of a monthly infusion.^61^

PLD was also compared to conventional doxorubicin as a first-line treatment for MBC in a multicenter study involving 509 patients.^62^ Results demonstrated that PLD provided comparable efficacy to doxorubicin (PFS = 6.9 *vs.* 7.8 months, respectively; HR = 1.00; 95% CI = 0.82–1.22; and OS = 21 *vs.* 22 months; HR = 0.94; 95% CI = 0.74–1.19), with reduced incidence of cardiotoxicity (HR = 3.16; 95% CI = 1.58–6.31; *P* < 0.001), myelosuppression (4% *vs.* 10%), and vomiting (19% *vs.* 31%) and alopecia (general, 20% *vs.* 66%; pronounced, 7% *vs.* 54%), although effects such as palmoplantar erythrodysesthesia (48% *vs.* 2%), stomatitis (22% *vs.* 15%) and mucositis (23% *vs.* 13%) were more frequently associated with PLD than with doxorubicin.

A large study across 143 centers in 19 countries was conducted to compare the use of combined PLD and docetaxel *vs.* docetaxel monotherapy for prolonging time to disease progression and incidence of cardiotoxicity in 751 women with advanced breast cancer who had relapsed at least 1 year after prior use of adjuvant or neoadjuvant anthracycline therapy.^63^ The results showed that treatment with PLD + docetaxel significantly improved median TTP from 7.0 to 9.8 months (HR = 0.65; 95% CI = 0.55–0.77; *P* < 0.001) and increased ORR from 26% to 35% (*P* < 0.001), while OS did not differ between the two groups (HR = 1.02; 95% CI = 0.86–1.22). The incidence of grade 3 or 4 adverse events was similar (78% *vs.* 72%), despite the observed higher incidence of palmoplantar erythrodysesthesia (24% *vs.* 0%) and mucositis/stomatitis (12% *vs.* 1%) with the PLD + docetaxel combination therapy. However, symptomatic cardiac events were reported in 5% and 4% of the treatment groups, demonstrating that PLD + docetaxel combination therapy improves clinical results compared to docetaxel monotherapy without increasing the risk of cardiotoxicity in this specific patient population.

More recently, the PELICAN study evaluated, between 2006 and 2010, the use of PLD *vs.* capecitabine as first-line therapy for MBC in 210 patients who were ineligible for endocrine therapy or trastuzumab with a median TTP as the primary endpoint.^64^ The results did not demonstrate superiority in the efficacy of PLD compared to capecitabine in patients with MBC, as reflected by the TTP (6.0 *vs.* 6.1 months; HR = 1.08; 95% CI = 0.76–1.54; *P* = 0.670) and OS (23.3 *vs.* 26.8 months; HR = 1.12; *P* = 0.530).

In terms of adverse events, patients receiving PLD had a higher incidence of leukopenia of any grade (38% *vs.* 17%; *P* = 0.002), alopecia (28% *vs.* 10%; *P* = 0.002), constipation (26% *vs.* 10%; *P* = 0.005), and stomatitis (40% *vs.* 17%; *P* < 0.001), while patients receiving capecitabine had higher rates of diarrhea (43% *vs.* 16%; *P* < 0.001), pulmonary embolism (6% *vs.* 0%; *P* = 0.040), and thromboembolism of any grade (17% *vs.* 2%). Serious adverse events were also more common in the capecitabine arm. Despite these findings, no statistically significant difference was observed between the two regimens in patients’ quality of life. The authors concluded that both PLD and capecitabine are active, effective, and relatively well-tolerated first-line treatment options for MBC.^64^

##### PEGylated liposomal doxorubicin for the treatment of multiple myeloma

A landmark multicenter study conducted in Greece evaluated the replacement of doxorubicin with PLD in the standard vincristine, doxorubicin, and dexamethasone (VAD) regimen as first-line therapy for multiple myeloma.^56^ The study enrolled 259 patients between 1999 and 2001. The results showed no statistical significance between the objective response rates (61.4% and 61.3% for patients treated with standard VAD and VAD with PLD, respectively). The mean TTP for responders was 23.93 months (95% CI = 16.92–30.94) for standard VAD and 24.30 months (95% CI = 16.76–31.84) for VAD with PLD (*P* = 0.580).

The hematological and non-hematological toxicities were mild and evenly distributed between the two treatment groups, except for baldness, which occurred more frequently with regular VAD, and palmoplantar erythrodysesthesia, which was more prevalent with VAD combined with PLD. The multicenter study demonstrated that both standard VAD and VAD with PLD can be administered to patients on an outpatient basis, ensuring equitable access to prompt intervention for numerous patients with symptomatic multiple myeloma.^56^

Another multicenter research involving 192 patients between 2001 and 2003 demonstrated that PLD as a component of the VAD regimen reduced the toxic effects of chemotherapy, such as grade 3/4 neutropenia or neutropenic fever (10% *vs.* 24%; *P* = 0.010). It significantly decreased the need for supportive care, with a lower incidence of sepsis, less antibiotic use, reduced need for central venous access (*P* < 0.001), and less alopecia (20% *vs.* 44%; *P* < 0.001), while maintaining comparable ORRs (44% *vs.* 41%), PFS (HR = 1.11; *P* = 0.690) and OS (HR = 0.88; *P* = 0.670) to conventional doxorubicin in newly diagnosed multiple myeloma. The findings highlighted an improvement in the clinical usefulness of the therapy, providing opportunities for transplantation.^57^

A robust investigation involving 646 patients from 123 centers between 2004 and 2006 evaluated the addition of PLD to standard therapy with bortezomib, a 26S proteasome inhibitor, in patients with relapsed or refractory multiple myeloma.^58^ The addition of PLD was able to increase the mean time to tumor progression from 6.5 to 9.3 months (*P* < 0.001; HR = 1.82; 95% CI = 1.41–2.35). The 15-month survival rate for PLD + bortezomib was 11% higher compared with bortezomib alone (76% *vs.* 65%; *P* = 0.030). The mean duration of response increased from 7.0 to 10.2 months (*P* < 0.001) with PLD + bortezomib. Conversely, the combined complete and partial response rates were not statistically significant (41% for bortezomib and 44% for PLD + bortezomib). In addition, grade 3/4 adverse events were more frequent in the combination group (80% *vs.* 64%), with an increased incidence of grade 3 or 4 neutropenia, asthenia, thrombocytopenia, diarrhea, fatigue, and erythrodysesthesia being observed palmoplantar. Despite these findings, the authors concluded that PLD + bortezomib is superior to bortezomib monotherapy for treating patients with relapsed or refractory multiple myeloma, representing an additional new standard of care for this group of patients.^58^

A secondary analysis of the same clinical study evaluated patients previously treated with thalidomide/lenalidomide who experienced therapeutic failure. The results revealed that the median TTP was significantly longer with PLD + bortezomib compared to bortezomib alone (270 days *vs.* 205 days; HR = 1.62; CI 95% = 1.08–2.41; *P* = 0.018), without increasing the incidence of grade 3/4 adverse events for patients exposed to prior treatment compared to those who were not previously treated.^59^ The median OS in the PLD + bortezomib cohort was 33 months (95% CI = 28.9–37.1), compared to 30.8 months (95% CI = 25.2–36.5) in the bortezomib monotherapy group (HR, 1.047; 95% CI = 0.879–1.246; *P* = 0.607). The study determined that, although PLD + bortezomib resulted in an extended TTP, long-term follow-up indicated no improvement in OS compared to bortezomib monotherapy in patients with relapsed and/or refractory multiple myeloma.^60^

##### Pegylated liposomal doxorubicin for the treatment of Kaposi's sarcoma

A multicenter (22 centers) phase III clinical trial conducted by the International Pegylated Liposomal Doxorubicin Study Group compared the clinical response of PLD *vs.* combination therapy with bleomycin and vincristine (BV) in 241 patients with AIDS-related KS (AIDS-KS) between 1993 and 1995.^54^ Treatment with PLD exhibited a higher combined response rate than BV (58.7% *vs.* 23.3%; *P* < 0.001), with a shorter response time (mean, 49 *vs.* 57 days). Furthermore, patients randomized to receive BV exhibited a higher likelihood of early treatment cessation due to adverse events (26.7% *vs.* 10.7%), resulting in a reduced completion rate of the full six treatment cycles (30.8% *vs.* 55.4%).

Both PLD and BV were linked to symptoms, such as anemia (18.2% *vs.* 15%), asthenia (11.6% *vs.* 13.3%), fever (15.7% *vs.* 25%), headache (12.4% *vs.* 8.3%), and nausea (11.6% *vs.* 16.7%). Grade 3 complications were less common. The treatment with BV was linked to a markedly increased occurrence of peripheral neuropathy (*P* < 0.001), while PLD treatment was more frequently related with neutropenia (*P* ≤ 0.001). The authors concluded that PLD proved to be an effective treatment for AIDS-KS, showing a stronger response and better patient tolerance compared to the BV combination, despite being more myelosuppressive.^54^

A phase III, multicenter (25 centers) randomized clinical trial was conducted between 1993 and 1994 to compare the efficacy and toxicity of PLD with standard chemotherapy with doxorubicin, bleomycin, and vincristine (DBV) in 258 patients with advanced KS AIDS-related.^38^ The results of this comparative study demonstrated the efficacy of PLD in patients with advanced AIDS-KS and its superiority to the DBV combination, with overall objective response rates of 45.9% (95% CI = 37–54%) for PLD compared to 24.8% (95% CI = 17–32%) for DBV (*P* < 0.001). Furthermore, the author emphasized that the superior results of PLD compared to a combination regimen containing conventional doxorubicin indicate that pegylated liposomal encapsulation may enhance the therapeutic effect of doxorubicin.

As for toxicities, the most common ones in DBV therapy for AIDS-KS were alopecia, neuropathy, nausea, and vomiting. Compared to the PLD treatment, the incidence of alopecia was four times higher in the DBV arm. In addition, no patient treated with PLD experienced grade 4 alopecia, whereas this adverse effect appeared in 6% of patients treated with DBV. Nausea and vomiting occurred twice as often in the DBV arm. Paresthesia and peripheral neuropathy, likely related to vincristine, appeared much more frequently in the DBV group. Ultimately, 37% of DBV patients discontinued their participation in the study because of an adverse event, compared to 11% for PLD patients (*P* < 0.001). Thus, the study demonstrated that PLD was more effective and less toxic than the standard DBV combination regimen for the treatment of AIDS-KS.^38^

A secondary analysis of the previous study evaluated the HQL of patients treated with PLD compared to standard DBV care.^55^ For definition purposes, HQL refers to measuring emotional, social, physical, psychological, and symptom outcomes resulting from the underlying illness as perceived by patients. The study concluded that PLD produced more benefits in HQL (with statistically significant improvements in energy/fatigue and pain, and potentially in overall health, social functioning, and general quality of life) compared to those treated with DBV, presenting itself as an enhanced palliative therapy than standard chemotherapy for this common complication secondary to AIDS.

##### PEGylated liposomal doxorubicin for ovarian cancer treatment

In a pioneering long-term follow-up study involving 413 patients with refractory or recurrent epithelial ovarian cancer at 104 centers in Europe, the US, and Canada, PLD treatment significantly prolonged survival compared with topotecan treatment, with an 18% reduction in the risk of death (median survival 62.7 *vs.* 59.7 weeks; HR = 1.216; CI 95% = 1.000–1.478; *P* = 0.050).^65^ The survival advantage was notably higher in individuals with platinum-sensitive illness, with a 30% decrease in mortality risk (median survival 107.9 *vs.* 70.1 weeks; HR = 1.432; 95% CI = 1.066–1.923; *P* = 0.017), being similar between groups for patients with platinum-refractory disease.

The Multicenter Italian Trials in Ovarian Cancer-2 (MITO-2) study tested whether PLD + carboplatin was more effective than standard first-line chemotherapy with carboplatin + paclitaxel for patients with advanced ovarian cancer, with mean PFS times of 19.0 *vs.* 16.8 months (HR = 0.95; 95% CI = 0.81–1.13; *P* = 0.580) and mean OS time of 61.6 *vs.* 53.2 months (HR = 0.89; 95% CI = 0.72–1.12; *P* = 0.320). The results did not demonstrate the superiority of the regimen containing the nanodrug and thus was not considered as standard first-line treatment for advanced ovarian cancer. Nevertheless, it can be regarded as an alternative to standard therapy.^68^

Between 2005 and 2007, a study by the Gynecologic Cancer Intergroup (GCIG), called Caelyx in Platinum Sensitive Ovarian patients (CALYPSO), which is considered the largest clinical trial conducted in refractory/recurrent ovarian cancer, enrolled 976 patients to test the efficacy and safety of the combination of PLD + carboplatin (comparator) compared to carboplatin and paclitaxel (standard).^66^ With a mean follow-up of 22 months, the PFS for the comparator arm was statistically superior to that of the standard arm (HR = 0.821; 95% CI = 0.72–0.94; *P* = 0.005), with a median PFS of 11.3 *vs.* 9.4 months, respectively. The nanodrug therapy also demonstrated a better therapeutic index, making it a more effective and less toxic alternative for patients who relapsed >6 months after platinum chemotherapy. This large-scale study resulted in numerous developments involving subsets in the years following the publication of the first results. Evaluating the safety of both therapies in patients aged ≥70 years, a higher therapeutic index and lower toxicity for PLD treatment were observed in older women with recurrent platinum-sensitive ovarian cancer.^67^ Furthermore, a sub-study demonstrated that the combination of PLD with carboplatin provided an attractive additional benefit over standard care by minimizing the risk of hypersensitivity reactions to the carboplatin-based regimen.^69^

In another publication, the CALYPSO study evaluated the treatment-related quality of life,^70^ and showed that the combination therapy with PLD was associated with statistically higher physical and global function scores at 3 months, although the differences are of modest clinical significance. However, these results showed that the best disease-related outcomes (e.g., PFS) of the previously described treatment were not achieved at the expense of poor quality of life.

Another CALYPSO-derived sub-study evaluated the effects of treatment with PLD + carboplatin *vs.* carboplatin + paclitaxel in a subset of 344 patients with partially platinum-sensitive ovarian cancer and showed that the experimental therapy exhibited a more favorable risk–benefit profile than standard therapy did, and should be considered as an effective treatment option for these patients (PFS = 9.4 *vs.* 8.8 months; HR = 0.73; 95% CI = 0.58–0.90; *P* = 0.004).^71^ Similarly, based on the CALYPSO trial, the efficacy of the treatments was evaluated in 259 patients with very platinum-sensitive ovarian cancer and showed that both treatments were equally effective for patients with this type of tumor (median PFS = 12.0 *vs.* 12.3 months; HR = 1.05; 95% CI = 0.79–1.40; *P* = 0.730; median OS = 40.2 *vs.* 43.9; HR = 1.18; 95% CI = 0.85–1.63; *P* = 0.330; ORR = 42% *vs.* 38%; *P* = 0.460). However, the favorable risk–benefit profile suggests that PLD + carboplatin is the ideal treatment of choice for these patients.^73^

The final assessment of the OS was published later to improve data accuracy. With a median follow-up of 49 months, no statistically significant difference was observed in the OS between the arms (HR = 0.99; 95% CI = 0.85–1.16; *P* = 0.940). The median survival in the experimental and standard arms was 30.7 and 33.0 months, respectively. No statistically significant difference was observed in the OS between arms when subgroups were stratified by age, body mass index, treatment-free interval, detectable disease, number of prior chemotherapy regimens, or performance status.^72^

More recently, a multicenter, open-label, randomized, phase III trial conducted in 159 academic centers in Germany, France, Australia, Austria, and the United Kingdom, evaluated the effectiveness of therapy with PLD + carboplatin + bevacizumab (experimental treatment) *vs.* gemcitabine + carboplatin + bevacizumab (standard care) in 682 patients with recurrent platinum-sensitive ovarian cancer between 2013 and 2015. The median PFS was 13.3 months (95% CI = 11.7–14.2) in the experimental group compared to 11.6 months (95% CI = 11.0–12.7) in the standard group (HR = 0.81; 95% CI = 0.68–0.96; *P* = 0.012). The median OS was 31.9 months (95% CI = 28.5–34.8) in the experimental group *vs.* 27.8 months (95% CI = 25.5–30.2) in the standard group (HR = 0.81; 95% CI = 0.67–0.98; *P* = 0.032). Based on these results, the regimen containing PLD + carboplatin + bevacizumab was considered a new standard treatment option for platinum-sensitive recurrent ovarian cancer.^74^

The extensive clinical evaluation of PLD, across multiple cancer types, such as multiple myeloma, KS, and ovarian cancer, highlights both its potential and limitations as a nanomedicine. The incorporation of PLD into standard chemotherapy regimens has demonstrated significant benefits in reducing toxicity and improving patient quality of life, which are critical considerations in cancer therapy. However, the OS benefits have been less consistent, highlighting the need for further innovation and optimization.

The evidence suggests that while PLD improves the therapeutic index of chemotherapy by reducing severe side effects, such as neutropenia, alopecia, and peripheral neuropathy, its impact on prolonging survival, particularly OS, is more variable.^72^ For instance, in multiple myeloma, PLD combined with bortezomib increased PFS but did not significantly enhance OS compared to bortezomib alone. Similarly, in ovarian cancer, while the CALYPSO study showed superior PFS with PLD + carboplatin compared to standard therapy, the final OS analysis did not demonstrate a significant difference. These findings imply that, while PLD is an effective tool for managing side effects and improving patients’ quality of life, it may not universally extend survival across all cancer types and treatment regimens.^69^, ^70^, ^71^, ^72^, ^73^

Future research should explore more sophisticated combinations of PLD with other targeted therapies, immunotherapies, or novel agents that could enhance its efficacy. Personalizing these combinations based on specific tumor biology and patient characteristics could potentially yield better outcomes, particularly in extending survival. Identifying biomarkers that predict patient response to PLD could refine its use, ensuring targeted application where it provides the greatest clinical benefit.^63^ The development of next-generation liposomal formulations or alternative nanocarrier systems that could further improve the delivery and efficacy of doxorubicin might address the limitations observed with PLD. Innovations that increase tumor specificity or enhance drug release mechanisms could potentially overcome some of the challenges observed in clinical outcomes. More extensive follow-up studies focusing on long-term survivorship and quality of life are necessary to understand the full impact of PLD-based therapies. This includes assessing late-onset toxicities, secondary malignancies, and overall functional status, which are critical for evaluating the true benefit of PLD in cancer management.^56^^,^^61^^,^^71^^,^^78^ As the evidence for this therapy grows, updating clinical guidelines may be necessary to better reflect its role in cancer treatment, including its use in combination therapies and protocols for patient selection, dosing, and monitoring.^31^^,^^53^^,^^58^^,^^74^

## Outlook section

The advancement of nanomedicines in oncology has marked a significant milestone in enhancing the efficacy and safety profiles of conventional chemotherapeutic agents. The integration of nanotechnology into cancer treatment, as exemplified by formulations, such as paclitaxel bound to albumin and PLD, has demonstrated improved therapeutic outcomes across various malignancies.^30^^,^^70^ This outlook section contextualizes the reviewed results, interprets their implications, and outlines future directions for the development and application of cancer nanomedicines, providing an in-depth exploration of the current research progress and significance in the relevant field.

The reviewed studies consistently showcase that nanomedicine formulations provide superior efficacy compared to their conventional counterparts. Paclitaxel bound to albumin has demonstrated increased OS, PFS, and ORR in cancers, such as NSCLC and pancreatic cancer. Similarly, PLD has shown comparable or improved efficacy in treating breast cancer, multiple myeloma, KS, and ovarian cancer while significantly reducing cardiotoxicity and other adverse effects commonly associated with traditional doxorubicin therapy.^58^^,^^62^^,^^63^ The improved safety profiles are attributed to the unique pharmacokinetic properties promoted by nanotechnology, which allows for controlled release and targeted drug delivery. These formulations reduce systemic toxicity, minimize off-target effects, and enhance patient quality of life, thereby offering more tolerable and effective treatment options.

Nanomedicines have addressed several unmet needs in oncology by overcoming limitations associated with traditional chemotherapies, such as poor solubility, rapid clearance, and non-specific distribution. The albumin-bound and liposomal encapsulation technologies have facilitated higher drug concentrations at tumor sites, improved penetration into tumor tissues, and provided options for patients who are refractory or have developed resistance to standard therapies.^6^^,^^13^^,^^19^^,^^30^ Moreover, these formulations have expanded therapeutic possibilities for specific patient populations, including older adults and those with comorbidities, by offering treatments with manageable toxicity profiles and convenient administration schedules, as evidenced by outpatient feasibility in multiple myeloma treatments.

The adoption of nanomedicine formulations has implications beyond clinical outcomes, impacting healthcare economics and resource utilization. Reduced adverse events and hospitalizations translate into decreased healthcare costs and improved allocation of medical resources. Additionally, the potential for outpatient administration reduces the burden on healthcare facilities and enhances patient convenience and compliance.^4^^,^^10^

## Future perspectives and prospects

Despite the successes highlighted in this work with existing nanomedicine formulations, several challenges remain to be addressed. A primary challenge is developing reproducible and affordable nanomedicines tailored to a specific cancer treatment on a large-scale, ensuring high loading capacity, long-term stability, and targeted delivery of the payload. Although nanomedicines are meant to increase drug distribution to certain areas, exact targeting remains challenging, and off-target effects may still be reported, thereby limiting their therapeutic outcomes. Several additional biological obstacles can also compromise the capacity of nanomedicines to efficiently treat the targeted cancer. The unique characteristics of nanomedicines further complicate adherence to established guidelines, leading to regulatory uncertainty and potential delays in licensing. To evaluate their long-term safety, nanomedicines could demand more thorough preclinical and clinical research. Future research should focus on incorporating stimuli-responsive and multifunctional nanoparticles to enhance specificity and therapeutic outcomes while minimizing adverse effects.

The integration of nanomedicines into personalized medicine approaches presents a promising avenue. Identifying and utilizing predictive biomarkers can help tailor nanomedicine therapies to individual patient profiles, maximizing efficacy, and minimizing toxicity. Personalized dosing regimens and combination therapies based on genetic and molecular tumor characteristics should be explored to enhance treatment precision.^7^^,^^13^^,^^18^

Conversely, exploring the efficacy of nanomedicines in a broader range of malignancies and in combination with other treatment modalities, such as immunotherapies and targeted agents, could further improve cancer treatment outcomes.^79^ Clinical trials investigating synergistic effects and optimal sequencing of therapies are warranted to establish comprehensive and effective treatment protocols.

Understanding and overcoming resistance mechanisms to nanomedicine therapies remain critical challenges. Research should focus on elucidating the molecular pathways contributing to resistance and developing strategies to counteract them, such as co-delivery of sensitizing agents or gene therapy approaches integrated within nanocarrier systems.

The complex nature of nanomedicine formulations necessitates streamlined regulatory frameworks and robust manufacturing processes to ensure quality, safety, and consistency.^23^ Collaboration between academia, industry, and regulatory bodies is essential to establish standardized guidelines and facilitate the translation of innovative nanomedicines from bench to bedside. Long-term safety data and post-marketing surveillance are crucial to fully understand the implications of nanomedicine therapies. Comprehensive monitoring and reporting systems should be implemented to track adverse events, rare toxicities, and overall patient outcomes over extended periods.^10^

Ensuring equitable access to advanced nanomedicine therapies is imperative. Efforts should be made to assess and improve the cost-effectiveness of these treatments, including exploring generic formulations and subsidized healthcare programs, to make them accessible to diverse patient populations globally.

## Conclusions

Nanotechnology offers new solutions for developing more effective and safer cancer treatments. The results of the clinical trials showed that (1) pegylated l-asparaginase can achieve a therapeutic effect similar to that of l-asparaginase with fewer applications due to its longer half-life as a result of surface modification with PEG; (2) paclitaxel bound to albumin improved therapeutic efficacy (i.e., better response rate in breast cancer, increased survival in pancreatic cancer in combination with gemcitabine, and longer PFS in NSCLC in combination with carboplatin), reduced infusion time, improved pharmacokinetics and eliminated the need for chemotherapy; reduced infusion time, improved pharmacokinetics, and eliminated the need for concomitant antihistamines and corticosteroids to prevent immune reaction to the solvent in the conventional paclitaxel formulation; and (3) PLD demonstrated a more efficient drug distribution pattern with higher tumor concentration and improved safety compared to conventional doxorubicin, resulting in reduced anthracycline-induced cardiotoxicity, better tolerability and adherence, and ultimately improved patient quality of life. These results suggest that nanomedicines, alone or in specific combinations, may represent an important modality capable of improving cancer treatment outcomes.

## Authors contribution

Micael N. Melo: Theoretical methodology, conceptualization, data search, and experimental data treatment and analyses, writing-review & editing; Ricardo G. Amaral: Theoretical methodology, conceptualization, data search and experimental data treatment and analyses, writing-review & editing; Lucas R. Melo de Andrade: Investigation and formal analysis, conceptualization, writing-review & editing; Patricia Severino: Investigation and formal analysis, conceptualization, writing-review & editing; Cristina Blanco-Llamero: Investigation and formal analysis, writing-review & editing; Luciana N. Andrade: Experimental methodology, investigation and formal analysis of data, conceptualization, writing–overviewed the manuscript, writing-review & editing; Eliana B. Souto: Experimental methodology, investigation and formal analysis of data, conceptualization, writing–overviewed the manuscript, writing-review & editing. All authors have read and agreed to the published version of the manuscript.

## Ethics statement

None.

## Declaration of generative AI and AI-assisted technologies in the writing process

The authors declare that generative artificial intelligence (AI) and AI assisted technologies were not used in the writing process or any other process during the preparation of this manuscript.

## Funding

This work was supported by the Coordination for the Improvement of Higher Education Personnel (*Coordenação de Aperfeiçoamento de Pessoal de Nível Superior* [CAPES]), National Council for Scientific and Technological Development (*Conselho Nacional de Desenvolvimento Científico e Tecnológico* [CNPq]), and the Sergipe State Research and Innovation Support Foundation (*Fundação de Apoio à Pesquisa e à Inovação Tecnológica do Estado de Sergipe* [FAPITEC]) and the University College Dublin research scheme fund 2024–2028 (No.82934-NP/R27885).

## Conflict of interest

The authors declare that they have no known competing financial interests or personal relationships that could have appeared to influence the work reported in this paper.
